# Supplementary material for: Effectiveness and Safety of Fufang Danshen Dripping Pill (Cardiotonic Pill) on Blood Viscosity and Hemorheological Factors for Cardiovascular Event Prevention in Patients with Type 2 Diabetes Mellitus: Systematic Review and Meta-Analysis
Source: Medicina (Kaunas). 2023 Sep 27;59(10):1730. doi: 10.3390/medicina59101730 (PMC10608055; doi:10.3390/medicina59101730)
Supplement: Supplementary file 1 [file medicina-59-01730-s001.zip › medicina-2601316-supplementary.pdf]

Supplement S1. Searching strategy

1. MEDLINE (PubMed) 2022.08.21

#1	((cardiotonic pills) OR (Danshen Dripping Pills) OR (fufang danshen dripping pill) OR (fufang danshen tablet) OR (Fufang Danshen Pian) OR (fufang danshen) OR (复方丹参滴丸))	162

2. CENTRAL (Cochrane)

#1	[mh "fufang danshen"] OR 'fufang danshen':ab,ti OR 'cardiotonic pills':ab,ti OR ' Danshen Dripping Pills':ab,ti OR 'Fufang Danshen Pian':ab,ti OR 'fufang danshen dripping pill':ab,ti OR '复方丹参滴丸'	63

3. EMBASE

#1	' fufang danshen'/exp OR 'fufang danshen pian':ab,ti OR 'cardiotonic pills'/exp OR ' Danshen Dripping Pills':ab,ti OR 'fufang danshen dripping pill'/exp OR 'cardiotonic pills':ab,ti OR 'cardiotonic pills'/exp OR 'fufang danshen tablet':ab,ti	211

4. CNKI

#1	(SU="复方丹参滴丸" OR AB="复方丹参滴丸" OR SU="fubang danshen-diwan" OR AB="fubang danshen-diwan" OR SU="cardiotonic pills" OR AB="cardiotonic pills" OR SU="fubang danshen" OR AB="fubang danshen" OR SU="fubang danshen dripping pills" OR AB="fubang danshen dripping pills" OR SU="fubang danshen tablet" OR AB="fubang danshen tablet" OR SU="Fufang Danshen Pian" OR AB="Fufang Danshen Pian") AND (AB="randomized controlled trial" OR AB="clinical trial" OR AB="random allocation" OR AB="randomly allocated" OR AB="random" OR AB="placebo" OR AB="	2,199

	随机对照研究" OR AB="临床随机对照试验" OR AB="随机对照" OR AB="临床试验")	

5. KCI

#1	심적환 AND (당뇨 OR Diabetics)	2

6. OASIS

#1	심적환 AND (당뇨 OR Diabetics)	3

7. RISS

#1	심적환 AND (당뇨 OR Diabetics)	9

Supplement S2. Characteristics of Included Studies

**NO.**	**First author, year**	**Country**	**Sample size**	**Age (yr), Mean±SD (range)**	**Sex (M:F)**	**Disease Duration**	**Disease classification**	**Diagnostic criteria**	**Treatment group intervention**	**Control group intervention**
**1**	Guo (2007)	China	**39:39**	T) 57.8 (46-69) C) 56.6 (44-70)	T: 47:31 C: 47:31	T) 9.6 (5.5–16.5) C) 9.4 (6.5–17.)	early DN	D1	CDDP + dietary control (0.8 g/(kg*d) with WM	Placebo + dietary control (0.8 g/(kg*d) with WM
**2**	Ye (2016)	China	50: 50	T) 67.36±2.01 C) 64.25±2.22	T) 24:26 C) 23:27	N/A	DM with asymptomatic myocardial ischemia	D2	CDDP	Conventional Therapy
**3**	Zhang (2009)	China	33:32	T) 58.0±11.2 C) 56.4±8.8	T) 21:12 C) 22:10	T) 10.4±5.8 C) 11.0±6.5	DR and DN	D1	CDDP 270 mg + Calcium hydroxybenzenesulfonate capsules 500 mg + Dietary control + Diabetic education + Protein 0.8 g (d*kg)+ insulin injection	Dietary control + Diabetic education + Protein 0.8 g (d*kg)+ insulin injection
**4**	Yang (2019)	China	50:50	T) 48.67±2.83 C) 48.15±2.22	T) 32:18 C) 31:19	T) 12.21±0.91 C) 2.62±0.95	DN		CDDP + irbesartan	irbesartan
**5**	JIA (2017)	China	50:50	T) 59.58±9.69 C) 58.06±13.01	T) 27:32 C) 30:20		DN	D3	CDDP + Valsartan	Valsartan
**6**	Zhao (2016)	China	37:37	T) 54.2±2.5 C) 57.8±1.1	T) 19:18 C) 21:16	T) 1–7 years C) 2–7 years	DM with asymptomatic myocardial ischemia		CDDP + β blockers + calcium blockers and other conventional treatment	β blockers + calcium blockers and other conventional treatment
**7**	Chen (2018)	China	60:60	T) 68.4±5.6 C) 68.5±5.5	T) 35:25 C) 33:27	T) 8.4±2.3 C) 8.5±2.2	DM Combined with Silent Myocardial Ischemia		CDDP	conventional treatment

**8**	Gu (2020)	China	41:41	T) 55.8±11.0 C) 56.5±10.5	T) 24:17 C) 19:22	T) 12.5±8.9 C) 13.1±8.5	DPN		CDDP	beloprost sodium
**9**	Huang (2005)	China	39:40	T) 57.2 ± 0.4 C) 56.9±0.5	T) 23:16 C) 22:18	-	DM with Nailfold capillary abnormalities	D1	CDDP + DM med (In case of HT patient`s Enalapril was prescribed.)	DM med (In case of HT patient`s Enalapril was prescribed.)
**10**	Yuan (2013)	China	47:47	*65 ± 5*	52 : 42	8.6 ± 1.5	early stage renal disease of type 2 DM	D4	CDDP + prostaglandin E1	prostaglandin E1
**11**	Jin (2015)	China	36 : 36	T) 68.2 ± 5.2 C) 65.1±4.8	T) 18:18 C) 17:19	6–14 y	DM complicated with asymptomatic myocardial ischemia		CDDP	conventional treatment methods, including hypoglycemic, lipid-lowering and nitrolipid drugs,
**12**	Lu (2017)	China	39 : 39	T) 43–68 C) 45–68	T) 26 : 13 C) 25 : 14	T) 6–13 y C) 6–12 y	DM and myocardial ischemia		CDDP	conventional treatment methods and the common drugs included hypoglycemic blockers.
**13**	Li (2015)	China	49 : 49	T) 68.25 ± 4.78 C) 67.85 ± 4.64	T) 28 : 21 C) 30 : 19	T) 5.33 ± 1.81 C) 5.12 ± 1.78	DM complicated with asymptomatic myocardial ischemia		CDDP	conventional treatment such as glucose lowering, blood pressure lowering, lipid lowering and p-receptor blockers
**14**	Lin (2017)	China	35 : 35	T) 57.2 ± 8.7 C) 58.5 ± 7.9	T) 24 : 11 C) 22: 13	T) 6.97 ± 5.23 C) 6.41 ± 5.64	DPN	D3 & D4	CDDP	oral Mecobalamin tablet
**15**	Wang (2016)	China	39 : 39	T) 55.9 ± 12.4 C) 56.4±12.3	T) 26 : 13 C) 27 :12	T) 9.5 ± 3.7 C) 9.2 ± 3.6	DM with asymptomatic myocardial ischemia and excluded the dysfunction of heart, kidney and	-	CDDP	routine treatment such as β Receptor blockers, hypoglycemic, lipid-lowering, calcium blockers, etc.

							other important organs and mental disorders			
**16**	Wang (2015)	China	39 : 39	T) 66.26 ± 0.74<br>C) 66.25 ± 0.75	T) 19 : 20<br>C) 20 : 19	T) 7.24 ± 0.15<br>C) 7.25 ± 0.14	DM complicated with asymptomatic myocardial ischemia	-	CDDP	routine treatment. (β Receptor blockers, calcium blockers and nitrates)
**17**	Yin (2017)	China	35 : 35	T) 57.6 ± 4.1<br>C) 58.8 ± 7.2	T) 19 : 16<br>C) 18 : 17	T) 8.9±1.6<br>C) 9.5 ± 2.5	DM and 24-hour urinary protein ≥ 30 mg	D4	CDDP	irbesartan
**18**	Bai (2008)	China	48 : 48	T) 68±4<br>C) 67±5	T) 27 : 21<br>C) 26 : 22	T) 8.9±1.5<br>C) 8.7±1.6	early DN	-	CDDP + irbesartan (ambovide) orally	irbesartan (ambovide)

DN: Diabetic Nephropathy; DPN: Diabetic Peripheral Neuropathy; DR: Diabetic Retinopathy, DM: Diabetes Mellitus.

D1) 1997 ADA Criteria, diagnostic staging method of diabetic nephropathy in Mogensen, Denmark; D2) Diagnosis of DM complicated with asymptomatic myocardial ischemia; D3) 2013 edition of the Chinese Diabetes Prevention and Control Guidelines; D4) WHO criteria for the diagnosis of type 2 diabetes.

Supplement S3. Details of Clinical trial Results of Included Studies

**First author, year**	**Treatment duration (Dosage /day)**	**Outcome Variable**	**Effective Results**	**Adverse Events**
Guo (2007)	8 Week, tid*10 pills	① TER<br>② Microalbumin test<br>③ Beta 2 Microglobulin test<br>④ FBG<br>⑤ TC<br>⑥ TG<br>⑦ HDL-C<br>⑧ LSR<br>⑨ HSR<br>⑩ PV<br>⑪ Fibrinogen	① 12.02±1.43*Δ vs 18.83±3.64<br>② 1.88±0.55*Δ vs 2.75±1.33 / 0.18±0.08*Δ vs 0.25±0.07<br>③ 6.14±1.70** vs 6.24±1.57<br>④ 5.42±0.66**ΔΔ vs 7.60±0.63<br>⑤ 1.96±0.81**ΔΔ vs 3.44±0.78<br>⑥ 1.55±0.25*Δ vs 1.43±0.23<br>⑦ 8.48±1.75**Δ vs 9.75±1.68<br>⑧ 4.68±1.50** vs 4.96±1.66<br>⑨ 1.80±0.79* vs 2.00±0.76<br>⑩ 2.90±0.78** vs 3.12±0.78	NR
Ye (2016)	10 pills (270mg)	① Hcy<br>② PA	① 6.04±1.24 vs 19.84±5.14<br>② 8.34±1.41* vs 11.21±4.14*	NR
Zhang (2009)	3 months, tid*10pills(270mg)	① TER<br>② LSR<br>③ HSR<br>④ PV<br>⑤ Platelet adhesion<br>⑥ Platelet aggregation rate<br>⑦ Sr<br>⑧ BUN<br>⑨ 24h Urine test	① Ameliorate 60.60*vs21.87 / Stable 36.36vs59.38 / Deteriorate 3.04**vs18.75<br>② 8.6±1.6 vs 10.6±1.7<br>③ 4.9±0.7 vs 5.7±0.8<br>④ 1.5±0.3* vs 1.7±0.3<br>⑤ 44±12* vs 53±10<br>⑥ 55±14 vs 64±11<br>⑦ 126±34* vs 102±28Δ<br>⑧ 6.8±1.7* vs 6.0±1.8Δ<br>⑨ 204±70* vs 149±80Δ	deteriorate (n=6)
Yang (2019)	1 month, tid*1 pills	① TER<br>② Serum albumin<br>③ Serum urea<br>④ Serum creatinine<br>⑤ ESR<br>⑥ WBV<br>⑦ PV	① 50 vs 37<br>② 31.11±3.26 vs 33.21±4.53<br>③ 11.25±1.24 vs 22.45±3.55<br>④ 322.45±12.01 vs 452.62±12.77<br>⑤ 9.23±0.21 vs 10.11±0.41<br>⑥ 4.12±0.13 vs 5.01±0.34<br>⑦1.66±0.21 vs 1.82±0.11	C) n=1<br>T) n=2

JIA (2017)	12weeks, tid*405mg	① ACR<br>② WBV<br>③ PV	① 6.06±3.56 vs 5.23±3.11(P=0.2209)<br>② 5.83±0.86 vs 5.47±0.87(P=0.0388)<br>③ 3.50±0.94 vs 3.60±0.82(P=0.5738)	NR
Zhao (2016)	Tid*10 pills	① TER<br>② PA<br>③ Hcy	① 87. 5 vs 57. 5<br>② 9. 24 ± 1. 57 vs 5. 45 ± 1. 38 *<br>③ 9. 86 ± 5. 38 vs 18. 29 ± 4. 71 *	NR
Chen (2018)	8 weeks, tid*10 pills	① TER<br>② Hcy<br>③ APN<br>④ WBC(10^9)<br>⑤ RBC(10^12)<br>⑥ Platelet(10^9)<br>⑦ TC<br>⑧ TG<br>⑨ LDL-C<br>⑩ HDL-C	① 50 vs 58<br>② 19.22±5.12 vs 11.23±4.45(P=0.000)<br>③ 4.53±1.05 vs 8.30±1.34(P=0.000)<br>④ 5.78±0.26 vs 5.88±0.25(P=0.034)<br>⑤ 4.56±0.07 vs 4.69±0.08(P=0.000)<br>⑥ 163.21±2.33 vs 165.33±2.32(P=0.000)<br>⑦ 5.63±1.17 vs 5.18±1.22(P=0.041)<br>⑧ 1.85±0.16 vs 1.40±0.21(P=0.000)<br>⑨ 3.86±1.42 vs 3.23±1.53(P=0.021)<br>⑩ 1.19±0.45 vs 1.68±0.47(P=0.000)	T) 10.00% n=6<br>C) 8.33% n=6
Gu (2020)	3 months, tid*10 pills	① TER<br>② median nerve MCV<br>③ median nerve SCV<br>④ common peroneal nerve MCV<br>⑤ common peroneal nerve SCV(m/s)<br>⑥ Abnormal foot pressure perception score<br>⑦ MnSi Symptom questionnaire scores<br>⑧ MnSi foot test score<br>⑨ VAS<br>⑩ WBV<br>⑪ PV<br>⑫ platelet aggregation rate<br>⑬ platelet adhesion rate	① 92.6 vs 77.8<br>② 47.34±1.91* vs 51.05±2.11*#<br>③ 46.47±1.93* vs 48.95±2.13*#<br>④ 46.99±1.93* vs 49.73±1.95*#<br>⑤ 49.33±2.11* vs 51.97±2.01*#<br>⑥ 6.2±2.5* vs 5.1±1.6*#<br>⑦ 6.7±1.6* vs 5.1±1.4*#<br>⑧ 3.4±1.3* vs 2.1±1.0*#<br>⑨ 2.6±1.3* vs 1.4±0.8*#<br>⑩ 11.4±1.6* vs 9.6±1.2*#<br>⑪ 2.0±0.5* vs 1.5±0.3*#<br>⑫ 26.9±5.5* vs 21.2±4.3*#<br>⑬ 41.7±7.0* vs 38.2±5.1*#	NR
Huang (2005)	3 weeks, tid*10 pills	① TC<br>② TG<br>③ LSR<br>④ HSR<br>⑤ PV	① 3.55±1.21** vs 6.35±1.21<br>② 1.38±0.86 ** vs 2.38±0.86 *<br>③ 8.21±1.47** vs 10.86±1.59<br>④ 3.02±0.19**vs 4.19±0.62 *<br>⑤ 1.63±0.16** vs1.87±0.19	mild headache (n = 2)

		⑥ Hct<br>⑦ ESR	⑥ 2.07±0.49 *vs 2.61±0.59<br>⑦ 40.96±2.92 vs 41.98±3.23	
Yuan (2013)	4 weeks, tid*10 pills	① Creatinine<br>② Blood urea nitrogen<br>③ 24-hour urine total protein<br>④ AER<br>⑤ HSR<br>⑥ LSR<br>⑦ PV<br>⑧ Fibrinogen	① 76±13 vs 79±10<br>② 5.5±1.4 vs 5.6±1.4<br>③ 0.12±0.06* vs 0.18±0.011<br>④ 61.5±9.8 vs 73.6±9.1<br>⑤ 6.1±0.6* vs 7.1±0.5<br>⑥ 10.0±3.2* vs 13.8±2.0<br>⑦ 1.7±0.6* vs 2.1±0.5<br>⑧ 2.9±0.3* vs 3.7±0.8	NR
Jin (2015)	1 year, tid* 10 pills(270mg)	① Hcy<br>② PA	① 8.29±1.42* vs 6.15±1.45*<br>② 11.2±4.8* vs 19.4±5.7*	NR
Lu (2017)	2 months, tid	① Hcy<br>② PA	① 11.3±4.2* vs 19.5±5.6*<br>② 8.31±1.42 vs 6.22±11.48	NR
Li (2015)	2 months, tid*10 pills	① Hcy<br>② APN	① 11.21±5.11* vs 19.25±5.12*<br>② 8.87±1.12* vs 6.17±1.89*	NR
Lin (2017)	3 months, tid*10 pills	① LSR<br>② HSR<br>③ PV<br>④ Hct<br>⑤ sensory nerve conduction velocity	① 17.25 ± 4.05 vs 20.48 ± 4.58<br>② 4.85 ± 2.16 vs 5.54 ± 2.78<br>③ 1.60 ± 0.17 vs 35.21 ± 3.95<br>④ 35.21 ± 3.95 vs 39.47 ± 4.12<br>⑤ 46.94 ± 13.37 vs 47.06 ± 13.95 / 55.83 ± 8.74 vs 56.74 ± 10.38 / 49.32 ± 10.8 vs 49.84 ± 11.54 / 45.05 ± 10.77 vs 45.85 ± 10.89	NR
Wang (2016)	8 weeks, tid*10 pills (270 mg)	① Hcy<br>② PA	① 11.3±4.2 vs 19.5±5.6 (P<0.05)<br>② 8.31±1.42 vs 6.22±1.48 (P<0.05)	NR
Wang (2015)	2 months, tid*10 pills (270mg)	① APN<br>② Hcy	① 8.32±1.51* vs 6.12±1.31<br>② 11.25±4.48* vs 20.15±5.09	NR
Yin (2017)	3 months, tid*10 pills	① Scr(c/μmol·L-1)<br>② BUN<br>③ Beta 2 Microglobulin test<br>④ 24h Urine test<br>⑤ HSR<br>⑥ LSR<br>⑦ PV	① 86.46 ± 21.35* vs 95.41 ± 26.45*<br>② 5.28 ± 1.27* vs 5.79 ± 1.35*<br>③ 371.67 ± 172.53* vs 521.52 ± 225.38*<br>④ 208.14 ± 21.36* vs 246.78 ± 25.76*<br>⑤ 6.07 ± 0.56* vs 6.92 ± 0.53*<br>⑥ 10.05 ± 0.53 vs 13.76 ± 0.32*	Dizziness (1 vs 3)<br>nausea, vomiting (1 vs 3)<br>lower limb pain (0 vs 2)

		⑧ Fibrinogen	⑦ 1.51 ± 0.25* vs 1.95 ± 0.41*<br>⑧ 2.68 ± 0.24* vs 3.55 ± 0.48*	diarrhea (1 vs 3)
Bai (2008)	16 weeks, tid*10 pills	① HSR<br>② LSR<br>③ PV<br>④ Fibrinogen<br>⑤ Hct<br>⑥ TC<br>⑦ TG<br>⑧ HDL-C<br>⑨ LDL-C<br>⑩ Creatinine<br>⑪ BUN<br>⑫ Serum creatinine clearance(ml/min)<br>⑬ 24h Urine test<br>⑭ Urinary albumin excretion rate	① 6·2±0·6* vs 7·2±0·5<br>② 10·1±3·2* vs 13·9±2·0<br>③ 1·7±0·6* vs 2.2±0.5<br>④ 2.9±0.3* vs 3.8±0.9<br>⑤ 0.38±0.05* vs 0.53±0.04<br>⑥ 4.8±0.6* vs 6.5±0.4<br>⑦ 2.45±0.28 vs 1.52±0.27*<br>⑧ 1.07±0.35 vs 1.11±0.26<br>⑨ 3.64±0.21* vs 4.02±0.45<br>⑩ 75±13 vs 79±10<br>⑪ 5.7±1.4 vs 5.6±1.4<br>⑫ 83.0±26.8 vs 83.8±24.6<br>⑬ 120±60* vs 190±50*<br>⑭ 61.4±9.8* vs 73.5±9.1*	No side effects were reported

Hcy: Homocysteine (μmol/L); HSR: high shear rate viscosity (mpa/s); LSR: low shear rate viscosity (mpa/s); PA: plasma adiponectin (mg/L); PV: plasma viscosity (mpa/s); WBV: whole blood viscosity (mpa/s); 24 h Urine protein: 24-hour quantitative determination of urinary protein (m/mg); 24 h Urine test: 24-hour Urine quantitative protein (mg/24 h).

Unit of each outcome variables: ACR (mg/mmol); AER (μg/min); APN (mg/L); Beta 2 Microglobulin test (mg/L); Blood urea nitrogen (mmol/L); BUN (mmol/L); Creatinine (μmol/L); FBG (mmol/L); Fibrinogen (g/L); Hcy (μmol/L); HDL-C (mmol/L); LDL-C (mmol/L); Microalbumin test (μg/min); PA (mg/L); Platelet adhesion (%); Platelet aggregation rate (%); Plasma viscosity (mpa/s); Serum albumin (g/L); Serum creatinine (mmol/L); Serum creatinine clearance (mL/min); Serum urea (mmol/L); Sr (μmol/L); TC (mmol/L); TER (%); TG (mmol/L); Urinary albumin excretion rate (μg/min); Whole blood viscosity (mpa/s); 24-hour urine total protein (g/24 h).

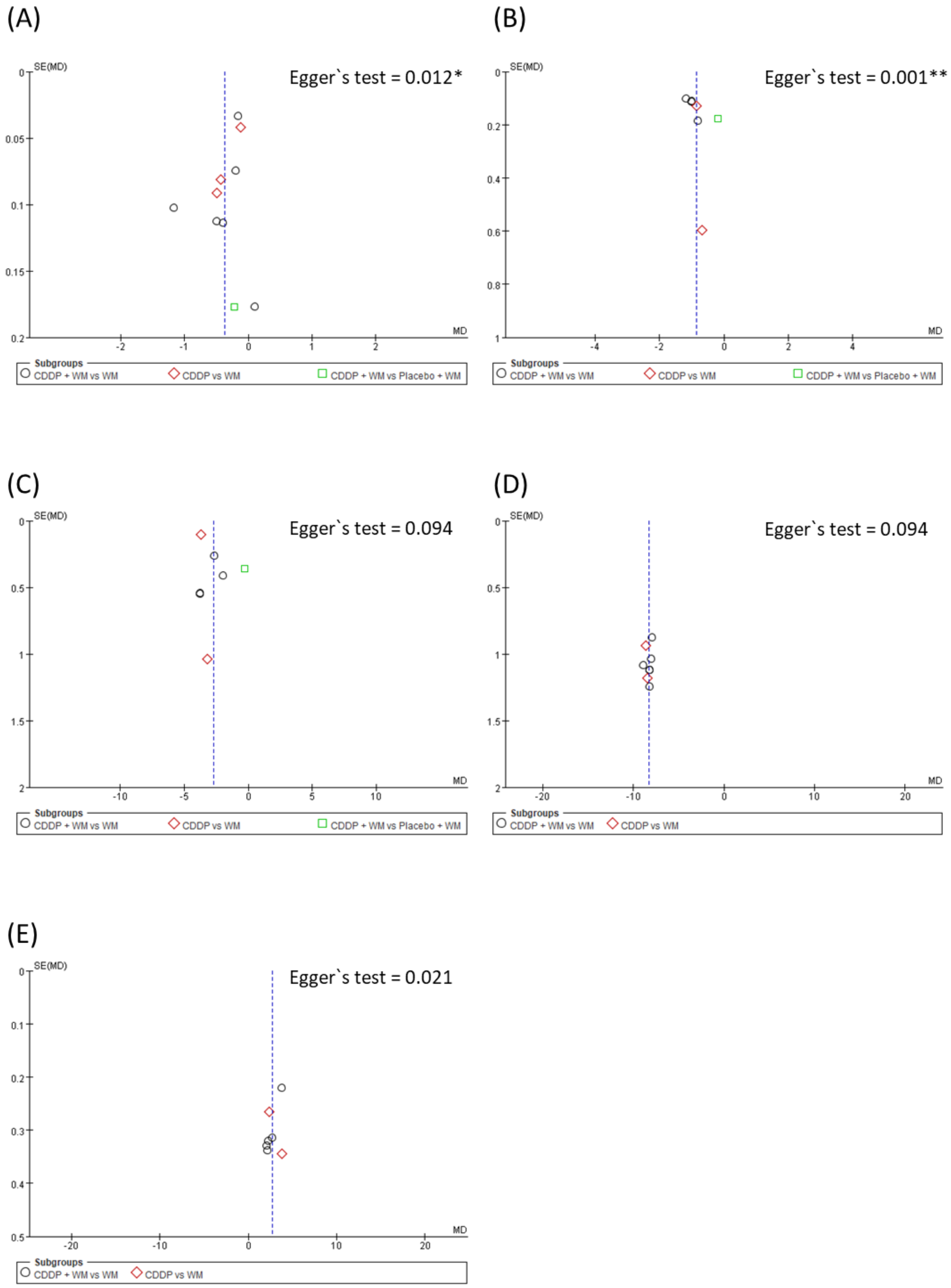

Supplement S4. Funnel plot illustrating publication bias. (A) Plasma viscosity, (B) high shear rate, (C) low shear rate, (D) homocysteine, and (E) plasma adiponectin.

Supplement S5. GRADE assessment of included studies.

Certainty assessment							№ of patients		Effect		Certainty	Importance
№ of studies	Study design	Risk of bias	Inconsistency	Indirectness	Imprecision	Other considerations	CDDP	Control	Relative (95% CI)	Absolute (95% CI)		
**Plasma viscosity**												
10	randomized trials	not serious[a]	serious[b]	not serious	not serious	none	417	417	-	MD **0.37 lower** (0.54 lower to 0.19 lower)	⨁⨁⨁◯ Moderate	
**High Shear Rate**												
7	randomized trials	not serious	serious[b]	not serious	not serious	none	276	276	-	MD **0.86 lower** (1.08 lower to 0.63 lower)	⨁⨁⨁◯ Moderate	
**Low Shear Rate**												
7	randomized trials	not serious	not serious	not serious	not serious	none	276	276	-	MD **2.74 lower** (3.77 lower to 1.72 lower)	⨁⨁⨁⨁ High	
**Homocysteine**												

Certainty assessment							№ of patients		Effect		Certainty	Importance
№ of studies	Study design	Risk of bias	Inconsistency	Indirectness	Imprecision	Other considerations	CDDP	Control	Relative (95% CI)	Absolute (95% CI)		
8	randomized trials	not serious	not serious	not serious	not serious	none	349	349	-	MD **8.32 lower** (9.05 lower to 7.58 lower)	⨁⨁⨁⨁ High	
**Plasma Adiponectin**												
7	randomized trials	not serious	not serious	not serious	not serious	none	310	310	-	MD **2.72 higher** (2.13 higher to 3.32 higher)	⨁⨁⨁⨁ High	
